# Low birth weight may increase body fat mass in adult women with polycystic ovarian syndrome

**Published:** 2016-05

**Authors:** Sonia Minooee, Fahimeh Ramezani Tehrani, Parvin Mirmiran, Fereidoun Azizi

**Affiliations:** 1 *Reproductive Endocrinology Research Center, Research Institute for Endocrine Sciences, Shahid* *Beheshti University of Medical Sciences, Tehran, Iran.*; 2 *Nursing and Midwifery School,* *Alborz University of Medical Sciences, Karaj, Iran.*; 3 *Obesity Research Center, Research Institute for Endocrine Sciences, Shahid* *Beheshti University of Medical Sciences, Tehran, Iran.*; 4 *Endocrine Research Center, Research Institute for Endocrine Sciences, Shahid* *Beheshti University of Medical Sciences, Tehran, Iran.*

**Keywords:** *Body fat mass*, *Body lean mass*, *Birth weight*, *Poly cystic ovarian syndrome*

## Abstract

**Background:**

Women engaged with polycystic ovarian syndrome (PCOS), as the commonest endocrine disorder, are known to have a specific type of adiposity. Birth weight is among different contributors reported to be responsible for this diversity.

**Objective:**

We aimed to compare the relation between birth weight and body fat mass (BFM)/ body lean mass (BLM) in PCOS and their age and body mass index (BMI) matched normal controls.

**Materials and Methods:**

In this case-control study, a total number of 70 reproductive aged women, diagnosed with PCOS and 70 age- BMI matched healthy women without hirsutism and/or ovulatory dysfunction were recruited., control group had no polycystic ovaries in ultrasonographic scans. A detailed history of birth weight was taken and was divided into the following categories: <2,500 (low birth weight, LBW) and 2,500-4,000 (normal birth weight; NBW).

**Results:**

Results showed that LBW prevalence was higher in women with PCOS than in controls (19.3% (27) vs. 15.7% (22)). Also body fat and lean mass (BFM, BLM) have increased in adult women with PCOS who were born underweight compared to their normal (19.8±9.05 vs. 12.9±4.5, p=0.001 and 48.9±6.9 vs. 43.2±5.8, p=0.004 respectively).

**Conclusion:**

Fetal birth weight influences on the adulthood obesity, BFM and BLM. This impact is different among women with and without PCOS.

## Introduction

The etiology of Polycystic Ovarian Syndrome (PCOS) as the commonst endocrine disorder in reproductive aged women is not clear, although, the genetic and environmental factors are known to have an important role (1, 2). Recently numerous studies have concentrated on fat quantity and distribution among PCOS women (3-5). Patients with syndrome are at higher risk of global adiposity and increased visceral adipose tissue, not only in obese PCOS women but also in overweight and normweight women, which puts these groups at a greater risk of consequent obesity, insulin resistance, type 2 diabetes mellitus and cardiovascular disease (3, 6-8).

Data about body composition, mainly body fat mass (BFM) and body lean mass (BLM) in PCOS women are few and contradictory (9). Early-life factors such as fetal adipose tissue and birth weight (BW) are supposed to be associated with the development of adulthood obesity and BFM (10). Evidences show that higher BW does not necessarily demonstrate increased later adiposity (11, 12). Since low birth weight (LBW), could program a higher BFM, BW as an indicator of intrauterine growth, may play an important role in the later obesity (13, 14). PCOS patients tend to have higher BFM and lower BLM than healthy women, but so far the effects of underlying early life factors, like BW, on later body composition of women with PCOS have not been totally investigated (15).

We aimed to compare the relationship between birth weight and body composition in PCOS women and normal controls.

## Materials and methods

**Subjects and study design**

In this retrospective case-control study which was performed during March 2013 to March 2015, a total of 70 reproductive aged women referred to Reproductive Endocrinology Research Center were recruited for the purpose of present study. The Rotterdam criteria has been used for PCOS diagnosis (at least two of these three criteria: oligo- and/ or anovulation; hyperandrogenism and ultrasound criterion of PCO) (16). The study protocol was approved by the Medical Ethics Committee of the Research Institute for Endocrine Sciences of Iran. Written informed consent was obtained from all participants.

A detailed medical history was obtained on menstrual dates and regularity, hirsutism, acne and reproductive history was collected via standardized questionnaire (17). Weight was measured to the nearest 0.1 kg on a calibrated beam scale. Height and waist circumference (WC) were measured to the nearest 0.5 cm with a measuring tape. Waist was measured midway between the lower rib margin and the iliac- crest at the end of gentle expiration. Hirsutism was assessed by the main study investigator (F.R.T). Ovulatory dysfunction was defined using information on time intervals, cyclist and total number of menstrual cycles per year. A total of 70 women aged 18-45 yrs, without polycystic ovaries by ultrasonography and also without hirsutism and/or ovulatory dysfunction, by history and physical examination that refereed for annual gynecologic exam formed our ovulatory non-hirsute pool for selection of controls. The exclusion criteria were pregnancy, menopause, hyperprolactinemia, thyroid dysfunction, use of hormonal drugs and previous history of surgeries like hysterectomy or bilateral oophorectomy.

We frequency-matched our control subjects with PCOS cases based on age and BMI levels; we subdivided the subjects into three age subgroups of less than 25, 25-30 and over 30 year age groups, and then into BMI subgroups of less than 25, 25-30 and over 30 kg/m^2^ groups. So, nine groups were created. Then, participants with similar age and BMI were put into same subgroups. A detailed history of birth weight was taken; Birth weight was treated as nominal variable and divided into following categories: <2,500 (LBW) and 2,500-4,000 (NBW). Since the categorizations are not related to adulthood weight, wherever a weight classification is mentioned in the study, birth weight subgroups are meant.

**Evaluation of body composition**

Body composition was assessed using Body stat ® Body manager (Bodystat Ltd., Douglas, United Kingdom, serial no.310110) for all the study participants. The device output measured the total body fat mass, lean mass body water, dry lean weight (%), wellness marker and basal metabolism rate. In body composition analysis, body weight is the sum of BFM and LBM, which is composed of Dry Lean Mass and Total Body Water. Total Body Water is the sum of intracellular extracellular water Cells integrity can be characterized by the ratio between measured impedance values at 50 kHz and 5 kHz. This ratio is also called the wellness marker. All scans were done by a single trained technician according to manufacturer’s guidelines. 

**Statistical analysis**

All data are expressed as mean±SD. Differences between groups were compared by independent samples t-test. A one way analysis of covariance (ANCOVA) was carried out to model the associations between BFM, BLM and variables of age, BMI, WC and birth weight. ANCOVA is an extension of ANOVA that allows for the possible effects of covariates, such as WC on the response variable. We used BW as a factor, WC as a covariate and BFM and BLM as the response variables. P<0.05 was considered statistically significant. All statistical procedures were run on SPSS software (Statistical Package for the Social Sciences; SPSS Inc., Chicago, IL, USA).

## Results

In the present study, PCOS women were slightly younger (30.2±6.1 vs. 30.8±5.8 yrs), had lower BMI (22.8±6.4 vs. 23.09±2.8 kg/m^2^) and higher but not significant WC than non PCOS subjects (79.8±8.1 vs. 78.7±8.5 cm). Baseline characteristics of the study groups are shown in table I. Comparing fat and lean mass in the birth weight subgroups of women with PCOS, showed that BFM was not significantly different between LBW and NBW subjects (p=0.6). Regarding BLM, there was a significant difference between LBW and NBW subgroups (p=0.04) (Table II). 

**Figure 1:**
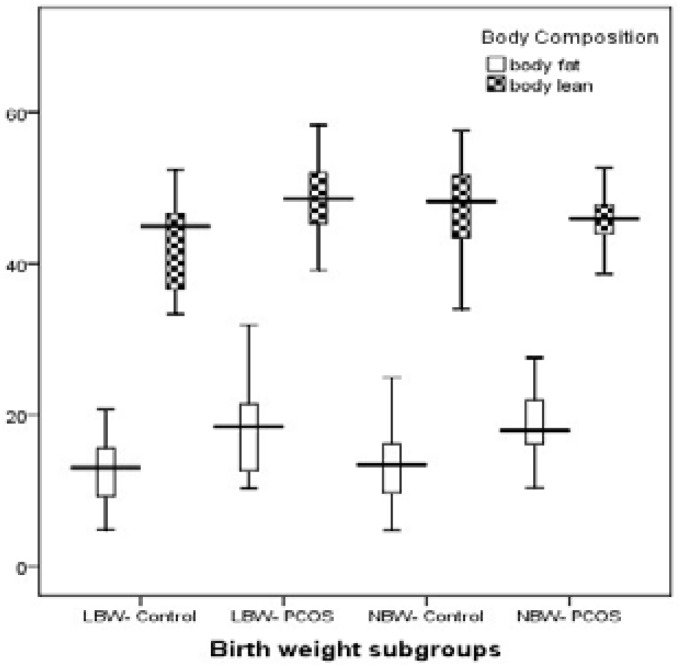
Summary of body fat and lean mass comparison between the PCOS and control subgroups.

Likewise, it was demonstrated that LBW subjects had lower BFM and BLM than NBW subjects in control group. We showed that both BFM and BLM is increased in adult PCOS patients born underweight than healthy women (19.8±9.05 vs. 12.9±4.5, p=0.001 and 48.9±6.9 vs. 43.2±5.8, p=0.004 respectively). Regarding fat mass, a similar finding was observed in the normal weight category (19.05±4.7 vs. 13.4±5.06, p=0.001). Findings for the main body composition elements (BFM, BLM), which was shown in table are summarized in Figure 1. Overall, it was stated that LBW neonates, who develop PCOS later in life, have increased body fat and lean mass than their non-PCOS counterparts. 

In a corrected model, ANCOVA test adjusted for WC, revealed that among the control group, although there was no association between BW and future fat mass (p=0.7), but there was a significant relation with lean mass (0.01). Among PCOS subjects, no association was observed between BW and lean mass but a significant relationship was detected with fat mass (p=0.1, p=0.01 respectively).

**Table I:** Baseline characteristics of the study participants (n=70

	**PCOS **	**Control **	**p- value**
LBW (%)*	27 (19.3)	22 (15.7)	-
NBW (%)*	43 (30.7)	48 (34.3)	-
Age (years)**	30.2 ± 6.1	30.8 ± 5.8	0.5
BMI (kg/m^2^)**	22.8 ± 6.4	23.09 ± 2.8	0.8
WC (cm)**	79.8 ± 8.1	78.7 ± 8.5	0.4
HC (cm)**	100.1 ± 6.8	98.1 ± 6.5	0.02
SBP (mmHg)**	101.1 ± 16.7	101.1 ± 9.3	0.01
DBP (mmHg)**	62.8 ± 11.7	65.4 ± 8.1	0.1
Parity**	1.4 ± 0.6	1.5 ± 0.7	0.5

PCOS: polycystic ovarian syndrome

LBW: low birth weight

NBW: normal birth weight

BMI: body mass index

WC: waist circumference

HC: hip circumference

SBP: systolic blood pressure

DBP: diastolic blood pressure

* Data are presented as frequency (percentage)

** Data are presented as mean±SD, using Student* t*- test.

**Table II:** Comparison of body composition elements between PCOS and control birth subgroups

	**PCOS**			**Control**		
	**LBW (n= 27)**	**NBW (n=43 )**	**p-value**	**LBW (n= 22)**	**NBW (n= 48)**	**p-value**
BFM (%)	19.8 ± 9.05	19.05 ± 4.7	0.6	12.9 ± 4.5	13.4 ± 5.06	0.7
BLM (%)	48.9 ± 6.9	45.8 ± 4.2	0.04	43.2 ± 5.8	47.3 ± 5.2	0.006
Body water (%)	35.4 ± 4.8	32.5 ± 2.7	0.006	33.7 ± 3.5	34.7 ± 3.6	0.3
DLW (%)	13.3 ± 2.4	13.3 ± 1.8	0.9	9.4 ± 2.6	12.5 ± 2.3	0.001
Wellness marker	0.88 ± 0.03	2.8 ± 6.3	0.05	1.1 ± 0.3	1 ± 0.2	0.3

Data are presented as mean (95% CI). Student *t*- test was performed to compare means of PCOS and control subgroups.

LBW: low birth weight

NBW: normal birth weight

BFM: body fat mass

BLM: body lean mass

DLW: dry lean weight

PCOS: polycystic ovarian syndrome

LBW: low birth weight

NBW: normal birth weight.

## Discussion

In the last decade emerging data have been made to prove that suboptimal intrauterine conditions program different health outcomes like body size, body composition, risk of type 2 diabetes mellitus, insulin resistance, dyslipidemia and obesity in the later life (18). In a process called “fetal programming”, early life factors may induce long-term effects on health (19). Barker *et al* was one of the first group to document a relationship between prenatal period and later disease like obesity or type 2 diabetes mellitus (20).

Studies have demonstrated that PCOS phenotype is modified by prenatal factors which are not clearly understood. Girls born with LBW will have accelerated childhood growth leading to anovulation, PCOS and metabolic syndrome (21). It is hypothesized that LBW and accelerated post natal growth may lead to a decrease in subcutaneous adipose tissue and provoke subsequent insulin resistance, hyperandrogenemia and PCOS (22). 

So far different studies have investigated body composition in women with PCOS, but the controversy still remains. Consistent with our observations, Carmina *et al* reported an increased BLM and comparable bone mass in PCOS patients to that of healthy women, similar to the findings of a Japanese study, that reported an increased regional lean mass in these patients (23, 24).

Nevertheless, in consistent with the above studies cited, Kirchengast and Huber showed a significantly higher BFM and lower BLM in lean PCOS women compared to weight matched controls, however this study included only a sample of 20 lean case and control women with a BMI of normal range (<25 kg/m^2^) (25). We should point out that, LBW alone has the potential to develop lower BLM and higher central adiposity measured by waist to hip ratio in normo-ovulatory population (26). 

Therefore, there are some reports indicating lower BLM, in the LBW cases, but similar to our findings, the combined status of LBW with PCOS (due to hyperandrogenemia) as a reverse effect and PCOS directly appears to be the main explanatory variable in higher fat and lean mass of patients who were born underweight. Since the association of BW and adulthood body weight is mainly due to the programming of greater lean mass, it seems that suboptimal intrauterine conditions result in a higher deficit in lean mass rather than in fat tissue (27). 

BW is a marker that reflects both fat mass and fat free mass and being overweight at birth alone, does not necessarily progress greater fat or lean mass in later adult life. Furthermore regarding other correlates of body composition except BW in PCOS women, data are restricted. One study showed an association between lean mass and fat parameters, insulin level and free androgen index but not total testosterone (23). 

In the present study, after adjustment for age, BMI, WC and BW, a significant positive correlation was present between fat mass and BW among women with PCOS. Overall, it is well-documented that an individual body composition is influenced by multiple genetic, nutritional and environmental factors (28, 29).

## Conclusion

To the best of our knowledge, this is the first study which investigated and compared the association between BW and body composition in PCOS patients with matched healthy women. However, the generalization of these findings requires replication of data in wider populations with different ethnic backgrounds.
